# The IL-25-dependent tuft cell circuit driven by intestinal helminths requires macrophage migration inhibitory factor (MIF)

**DOI:** 10.1038/s41385-022-00496-w

**Published:** 2022-03-14

**Authors:** Fumi Varyani, Stephan Löser, Kara J. Filbey, Yvonne Harcus, Claire Drurey, Marta Campillo Poveda, Orhan Rasid, Madeleine P. J. White, Danielle J. Smyth, François Gerbe, Philippe Jay, Rick M. Maizels

**Affiliations:** 1 Wellcome Centre for Integrative Parasitology, Institute of Infection, Immunity and Inflammation, University of Glasgow, Glasgow, UK; 2 Institute of Immunology and Infection Research, University of Edinburgh, Edinburgh, UK; 3 IGF, University of Montpellier, CNRS, Inserm, Montpellier, France; 4 Present Address: Lydia Becker Institute for Immunology and Inflammation, University of Manchester, Manchester, UK; 5 Present Address: Division of Cell Signalling and Immunology, University of Dundee, Dundee, UK

## Abstract

Macrophage migration inhibitory factor (MIF) is a key innate immune mediator with chemokine- and cytokine-like properties in the inflammatory pathway. While its actions on macrophages are well-studied, its effects on other cell types are less understood. Here we report that MIF is required for expansion of intestinal tuft cells during infection with the helminth *Nippostrongylus brasiliensis*. MIF-deficient mice show defective innate responses following infection, lacking intestinal epithelial tuft cell hyperplasia or upregulation of goblet cell RELMβ, and fail to expand eosinophil, type 2 innate lymphoid cell (ILC2) and macrophage (M2) populations. Similar effects were observed in MIF-sufficient wild-type mice given the MIF inhibitor 4-IPP. MIF had no direct effect on epithelial cells in organoid cultures, and MIF-deficient intestinal stem cells could generate tuft cells in vitro in the presence of type 2 cytokines. In vivo the lack of MIF could be fully compensated by administration of IL-25, restoring tuft cell differentiation and goblet cell expression of RELM-β, demonstrating its requirement upstream of the ILC2-tuft cell circuit. Both ILC2s and macrophages expressed the MIF receptor CXCR4, indicating that MIF may act as an essential co-factor on both cell types to activate responses to IL-25 in helminth infection.

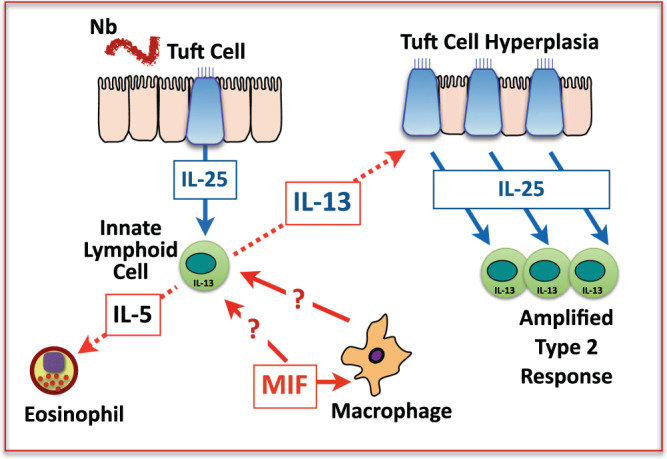

## Introduction

The cytokine macrophage migration inhibitory factor (MIF) was first identified in the 1960s by its ability to activate macrophages and arrest their motility in vitro^1,2^. MIF has more recently been classified as a member of the chemokine-like function family^3^ and as an atypical “multitasking” chemokine with a broad spectrum of targets^4,5^. MIF is prominent in the arena of type 1 immunity to microbial pathogens through effects such as enhancement of TLR4-dependent responses to LPS^6^, promoting inflammasome activation^7^and induction of inflammatory mediators from macrophages^8^, alongside antagonism of the immunosuppressive effects of glucocorticoids^9^.

MIF is constitutively expressed by epithelial cells, immune populations including T cells^10^, and macrophages themselves^11^. MIF is derived from an evolutionarily ancient gene family represented in both vertebrates and invertebrates^12^, and in the mammalian immune system acts primarily to activate innate pathways^13^. MIF-deficient mice show impaired clearance of *Salmonella typhimurium*^14^, *Streptococcus pneumoniae*^15^ and *Mycobacterium tuberculosis*^16^, but also improved survival when challenged with high dose LPS or enterotoxin B in models of sepsis. The pro-inflammatory character of MIF is also evident in human studies, in which polymorphisms controlling expression of this mediator influence development of pneumonia^17^, bacteremia^18^, inflammatory bowel disease^19^ and autoimmune hepatitis^20^. Furthermore, high serum MIF levels in septic shock are associated with poorer outcomes^21^.

In distinction to the many settings in which MIF drives classical IFN-γ/TNF mediated type 1 inflammatory pathways in response to microbial challenge, other data point to the ability of MIF to promote IL-4/IL-13-driven type 2 immunity. For example, MIF-deficient mice are protected from allergic airway inflammation in models of asthma^22–24^. At a cellular level, MIF is known to promote two key type 2 populations, alternatively activated macrophages (M2 cells) and eosinophils. We previously reported that MIF can synergise with IL-4 to induce bone marrow-derived macrophages to upregulate the canonical M2 products Arginase-1, Chil3 (Ym1) and RELMα^25^. MIF was also found to be important in alternative activation of tumour-associated macrophages^26^. In parallel, MIF is required for eosinophilic responses in airway^22,23^ and skin allergy^27^ models, as well as in mice infected with the trematode helminth *Schistosoma mansoni*^28^.

Type 2 immunity is critical for protection against helminth infections^29,30^, and hence the role of MIF in the anti-parasite response is of particular interest. MIF-deficient mice do not show greater susceptibility to *S. mansoni*, although development of eosinophil-rich granulomas is abated^28^. However, MIF^−/−^ mice have impaired immunity to the cestode tapeworm *Taenia crassiceps* associated with poorer macrophage cytokine responses^31^, while in recent work we found the same genotype cannot expel the gastrointestinal nematode *Heligmosomoides polygyrus*, even after an immunisation protocol that induces sterile immunity in wild-type mice^32^. Immunity to *H. polygyrus* requires both CD4^+^ T cells and type 2 innate lymphoid cells (ILC2s)^33,34^, leading to M2 macrophage activation^35–37^, which is delayed and diminished in MIF-deficient mice^32^.

In addition, the intestinal tissues play a critical role in worm expulsion^38,39^. Epithelial tuft cells detect the presence of helminths, releasing IL-25 which activates the ILC2 population that initiates type 2 immunity^40–45^. IL-4 and IL-13 feed back to amplify tuft cell differentiation, induce hyperplasia of goblet cells producing mucin and the anti-helminth protein RELMβ, and increase smooth muscle hypercontracility epitomising the “weep-and-sweep” response that eliminates luminal parasites^39,46^.

We have now examined the role of MIF in a more invasive nematode parasite *Nippostrongylus brasiliensis*, which enters through the skin, and transits to the lung before migrating to the small intestine. In both mucosal organs, it provokes a potent type 2 immune response, characterised by M2 macrophage and type 2 ILC2 proliferation and trafficking, as well as rapid parasite expulsion, unless deficient in key type 2 pathway components such as IL-4Rα or Stat6^47^, IL-25^48^ or tuft cells^49^. In this study we identify the wider impact of MIF signalling on ILC2 and macrophage activation in this acute model of nematode infection and demonstrate widespread downstream effects on epithelial cell effector populations, particularly goblet and tuft cells required for expression of protective immunity.

## Results

### MIF-deficient mice fail to expel the intestinal helminth *N. brasiliensis*

**Fig. 1:**
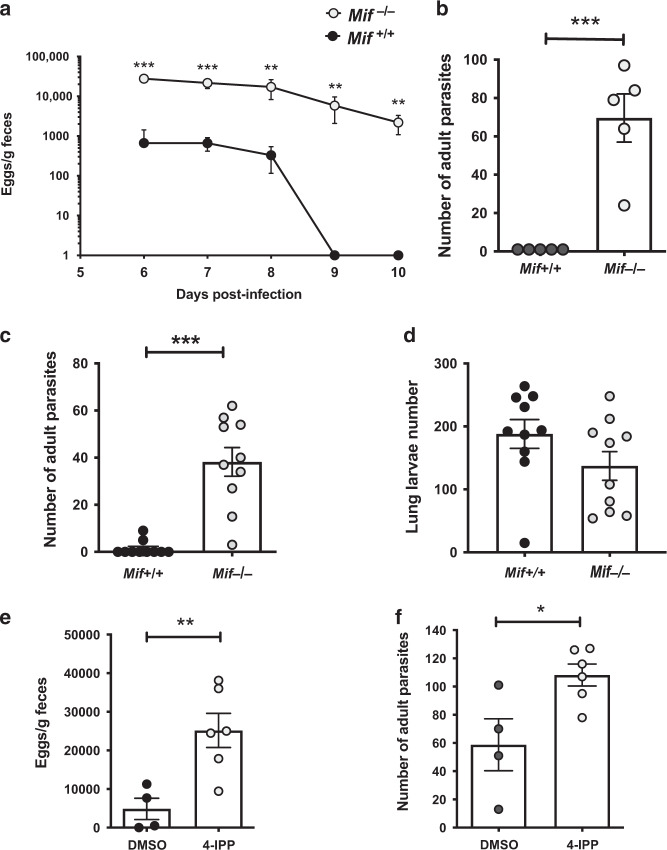
Impaired expulsion of *N. brasiliensis* by MIF-deficient and MIF-inhibitor- (4-IPP) treated mice. **a** MIF-sufficient (*Mif*^+/+^) and -deficient (*Mif*^−/−^) mice were infected with 250 *N. brasiliensis* L3 larvae of a mouse-adapted strain; fecal egg burdens were enumerated from day 6 post-infection. Data are representative of two independent experiments (*n* = 5 per group). **b** *Mif*^+/+^ and *Mif*^−/−^ mice were infected with 250 *N. brasiliensis* L3 larvae of a mouse-adapted strain and adult worm numbers counted at day 7. Data are representative of two independent experiments (*n* = 5 per group). **c** Adult *N. brasiliensis* worm count 6 days after infection with 400 *N. brasiliensis* L3 larvae of a rat-passaged strain in *Mif*^+/+^ and *Mif*^−/−^ mice. Data are representative of two independent experiments (*n* = 5 per group). **d** Recovery of larval *N. brasiliensis* from the lungs of *Mif*^+/+^ and *Mif*^−/−^ mice, 48 h after subcutaneous injection. Data are pooled from two independent experiments (*n* = 4–5 per group). **e**, **f** Impaired immunity to *N. brasiliensis* in wild-type BALB/c mice treated with the MIF inhibitor 4-IPP, measured as fecal egg burdens (**d**) and adult numbers counted on day 6 (**e**); *n* = 4 and 6 for DMSO and 4-IPP groups. Data are representative of three independent experiments. Data presented as arithemetic means ± standard errors and statistically analysed by multiple (**a**) or unpaired (**b**–**e**) *t* tests. *P < 0.05, **P < 0.01, ***P < 0.001.

We first examined if MIF deficiency altered the outcome of infection with the helminth parasite *N. brasiliensis*. Although a natural rat pathogen, *N. brasiliensis* normally persists in mice for 6–8 days before expulsion orchestrated by a strong type 2 immune response^47^. While wild-type BALB/c mice accordingly cleared parasites within 6–9 days of infection, parasite presence was prolonged in the small intestine of MIF-deficient (*Mif*^−/−^) animals with higher egg outputs (Fig. 1a) and a failure to promptly expel either of two strains of *N. brasiliensis*, a mouse-adapted strain, passaged in mice (Fig. 1b), or the conventional rat-passaged strain (Fig. 1c), respectively; indeed a small number of adult worms were still present in Mif^−/−^ mice 12 days post-infection (data not shown). The contrast between the two mouse genotypes is evident in increased egg production as early as day 6, indicating greater worm survival, enhanced fitness, or both. However, larval migration to the lung, which occurs in the first 24–48 h, is similar in both strains (Fig. 1d), indicating that the disparity is primarily in the intestinal immune response to infection.

We next tested whether a small molecule inhibitor of MIF, 4-IPP^50^ could recapitulate the MIF-deficient phenotype in wild-type mice. Indeed, this was found to be the case, as at day 6 of infection, 4-IPP treated mice excreted over 5-fold greater egg numbers than controls (Fig. 1e) and harboured nearly twice the number of adult worms (Fig. 1f).

### Restricted type-2 mesenteric lymph node responses in MIF-deficient mice

**Fig. 2:**
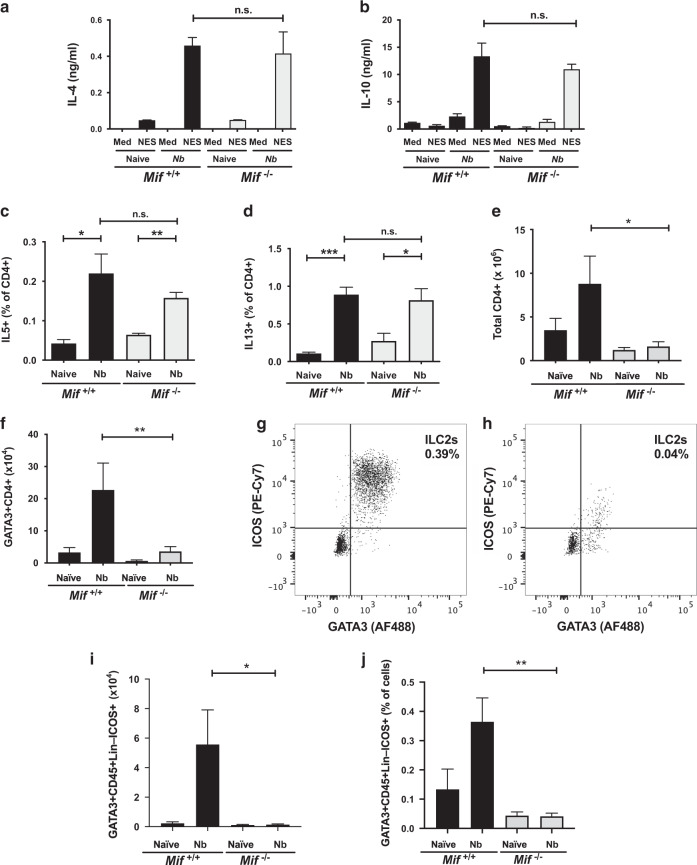
Expansion of type 2 effector cell populations is diminished in the MLN of MIF-deficient mice. MLN tissue was harvested from both *Mif*^+/+^ and *Mif*^−/−^ mice at d7 post *N. brasiliensis* infection and processed into single cell suspensions, for in vitro restimulation (**a**, **b**) and flow cytometry (**c**–**j**) using the gating strategy described in Fig. S3A. **a**, **b** IL-4 and IL-10 production by MLN cells from naive (*n* = 3) and d7 *N. brasiliensis*-infected *Mif*^+/+^ and *Mif*^−/−^ mice (*n* = 5), stimulated for 72 h with media or *N. brasiliensis* ES antigens (NES). Supernatants were assessed by ELISA. **c**, **d** Frequency of IL-5 and IL-13 expressing CD4^+^ T cells, as percentage of total CD4^+^ cells, from MLN of *N. brasiliensis*-infected *Mif*^+/+^ and *Mif*^−/−^ mice (*n* = 5) and naive controls (*n* = 3) identified by flow cytometry. Numbers of total CD4^+^ MLN T cells (**e**), and CD4^+^GATA3^+^ T cells (**f**) in *N. brasiliensis*-infected *Mif*^+/+^ and *Mif*^−/−^ mice (*n* = 7) and naive controls (*n* = 5–6) at day 7. **g**, **h** Exemplar flow cytometry plots of ICOS vs GATA3 staining of CD45^+^Lin^−^ MLN cells from *N. brasiliensis*-infected *Mif*^+/+^ and *Mif*^−/−^ mice at day 6. Percentages (**i**) and total numbers (**j**) of GATA3^+^CD45^+^CD4^−^Lin^−^ICOS^+^ILC2s among MLN cells from *N. brasiliensis*-infected mice (*n* = 4) and naive controls (*n* = 2–3) at day 6. Data in **a**–**d**, **i**, **j** are presented as arithmetric means and standard errors, and are all representative of two individually performed experiments; **e**, **f** represent pooled data from two independent experiments. Data were statistically analysed by unpaired *t* tests. *P < 0.05, **P < 0.01, ***P < 0.001.

As the adaptive Th2 response is a pre-requisite for immunity to *N. brasiliensis*^47^, we evaluated the IL-4 response of mesenteric lymph node (MLN) cells from naive and day 7 infected mice. When MLN cells were restimulated in vitro with the excretory-secretory antigens of adult parasites (NES), IL-4 levels were not significantly different between MIF-sufficient and -deficient mice (Fig. 2a). In the same system, it was also noted that MIF-deficient mice did not express greater amounts of IL-10, arguing that their enhanced susceptibility was not due to over-production of this immune suppressive cytokine (Fig. 2b), while no production of IFNγ was observed from cells of either genotype (data not shown). Furthermore, intracellular staining and flow cytometry of CD4^+^ MLN cells for further type 2 cytokines IL-5 and IL-13 confirmed that the strong Th2 bias of the anti-helminth response was intact in the absence of MIF (Fig. 2c, d).

However, while the quality of the response on a per-cell basis was similar in the two genotypes, we observed a striking quantitative difference, with markedly fewer total MLN cells in *Mif*^−/−^ mice than wild-type (1.6 × 10^5^ v 8.9 × 10^5^, Fig. 2e), mirrored by a paucity of CD4^+^GATA3^+^ Th2 cells (Fig. 2f) arguing that the overall intensity of the type 2 cytokine response is significantly diminished in the absence of MIF. Indeed, when we collected peritoneal lavage fluid from infected mice, levels of type 2 cytokines (which derive from both adaptive and innate cells) were markedly lower in the MIF-deficient animals (Fig. S1). We also assessed ILC2 responses in MLNs from naive and infected mice, defined as lineage-negative (Lin^−^) CD45^+^ICOS^+^GATA3^+^ cells; both the percentages (Fig. 2g–i) and total numbers (Fig. 2j) were greatly reduced in MIF-deficient mice, with no increase apparent following infection.

### MIF influences type 2 responses in the tissues

**Fig. 3:**
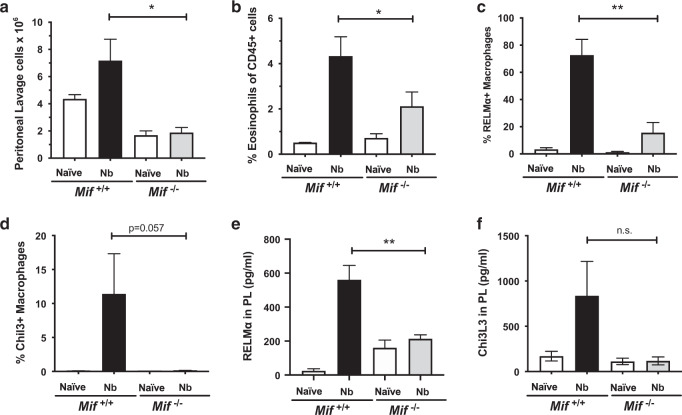
Concomitant with diminished Th2 and ILC2 responses, MIF deficiency leads to impaired eosinophil recruitment and alternative macrophage polarisation in the peritoneal cavity. Mice of both genotypes were infected with 400 L3 *N. brasiliensis* larvae by s.c. injection, and samples recovered at day 6 for cellular and ELISA analysis, using the gating strategy described in Fig. S3B. **a** Total peritoneal lavage cells recovered from infected mice (*n* = 8) and naive controls (*n* = 7), combined data from two independent experiments. **b** Siglec-F^+^ eosinophils recovered from infected mice (*n* = 5) and naive controls (*n* = 3) enumerated by flow cytometry. Alternatively activated CD11b^+^F4/80^+^ macrophages evaluated by expression of RELMα (**c**), and Chil3/Ym1 (**d**) in samples from infected mice (*n* = 5) and naive controls (*n* = 3), enumerated by flow cytometry. **e**, **f** Concentrations of RELMα (**j**) and Chil3/Ym1 (**k**) in the peritoneal lavage fluid measured by ELISA, in samples from infected mice (*n* = 3–4) and naive controls (*n* = 3). Data are presented as arithmetic means and standard errors, and **b**–**f** are representative of at least two individually performed experiments; data were statistically analysed by unpaired *t* tests. *P < 0.05, **P < 0.01*.*

We next turned attention to the innate immune cell populations in other tissues responding to *N. brasiliensis*, examining first cells from the peritoneal cavity, which show rapid activation during intestinal helminth infestations. We found cellular infiltration is sharply diminished in the absence of MIF (Fig. 3a) and the population present in infected MIF^−/−^ mice contains fewer eosinophils (Fig. 3b), or alternatively activated macrophages expressing the markers RELMα (Fig. 3c), and Chil3 (Ym1, Fig. 3d). The diminished RELMα and Chil3 responses were further reflected in soluble proteins present in peritoneal lavage measured by ELISA (Fig. 3e, f).

Responses of *N. brasiliensis*-infected mice were also examined in the lung, through which larval parasites transit between 24 and 48 h post-infection. Bronchoalveolar lavage (BAL) at 72 h post-infection revealed an inflammatory expansion only in the MIF-sufficient animals (Fig. S2A), with an absence of eosinophils from *Mif*^−/−^ airways (Fig. S2B) and fewer macrophages (Fig. S2C) with expression of RELMα only in wild-type mice (Fig. S2D). Baseline levels in naive animals, however, were broadly similar in all animals indicating no requirement for MIF in the steady state.

### Impaired type-2 epithelial responses in MIF-deficient mice

**Fig. 4:**
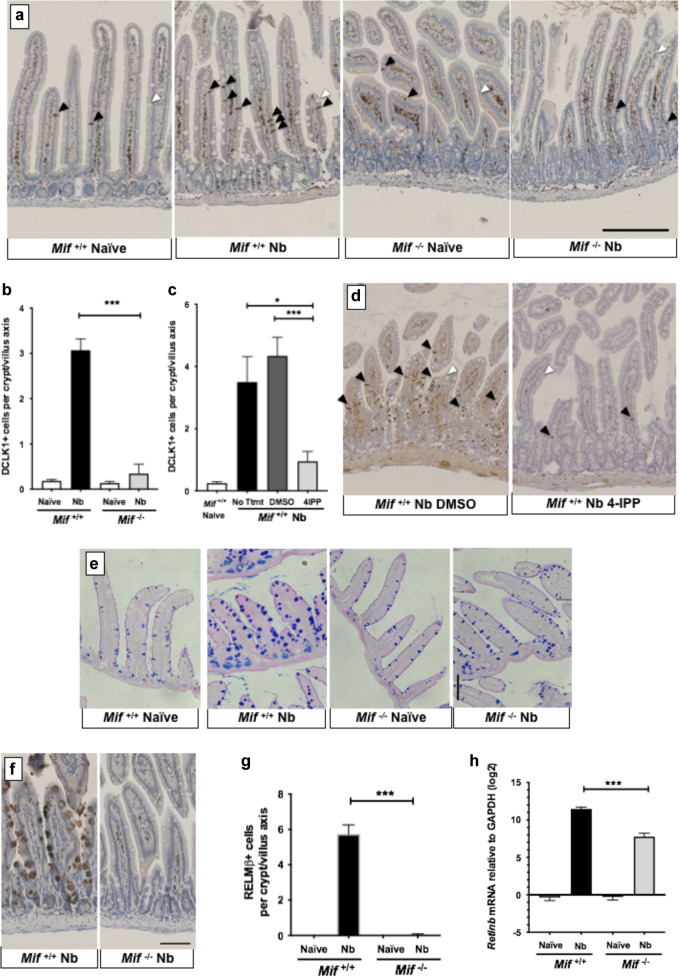
Intestinal epithelial responses to *N. brasiliensis* infections are aberrant in MIF-deficient mice. *Mif*^+/+^ and *Mif*^−/−^ mice were infected with 400 *N.brasiliensis* larvae by s.c. injection and intestinal tissues harvested 6 days later, staining paraffin-embedded sections for Dclk1 and RELM-β, and performing RT-PCR for mRNA. **a** Staining of small intestinal tissue sections of naive and infected mice with anti-Dclk-1 antibody. Neuronal cells within the lamina propria are stained in all samples. Tuft cells within the epithelial layer are indicated with black arrowhead. Examples of goblet cells indicated with white arrowhead. **b** Enumeration of Dclk1^+^ cells per crypt/villus axis (CVA), based on a minimum of 100 crypt/villus units, collected from three different individual mice for each group. **c**, **d** Inhibition of tuft cell response by the 4-IPP inhibitor. 1 mg 4-IPP or DMSO vehicle were administered on days 0, 2 and 4, with tissues recovered at day 6 following infection. Small intestinal tissues were taken for Dclk1 staining per crypt/villus axis, based on a minimum of 100 crypt/villus units from 3 to 6 individual mice for each group (**c**); examplar staining of control 4-IPP treated tissues are shown (**d**) with arrowheads as above. **e** Staining of Goblet cells in tissues of naive and infected mice with Periodic Acid-Schiff’s stain. **f** Staining of small intestinal tissue sections with anti-RELMβ antibody. **g** Enumeration of RELMβ^+^ cells per CVA, based on a minimum of 100 crypt/villus units from 3 to 4 individual mice for each group. **h** *Retnlb* (RELMβ) gene expression quantified by RT-PCR relative to GAPDH mRNA (log_2_ scale); data represent combined values from two experiments with a total of 7–8 mice per group. Data are presented as arithemetic (**b**, **c**, **g**) and geometric (**h**) means and standard errors, and are representative of at least two individually performed experiments, except **e** which was a single observation; data were statistically analysed by unpaired *t* tests. *P < 0.05, **P < 0.01, ***P < 0.001.

The intestinal epithelial cell layer is now recognised as an integral and essential participant in the type 2 response to helminths^38,39^. Immunity to *N. brasiliensis* requires tuft cell production of IL-25 driving IL-13-dependent hyperplasia^40–42^, concomitant with goblet cell expansion of mediators such as RELMβ. To evaluate responses in the epithelium, we employed mRNA expression and immunohistology, as following infection epithelial cells show very poor viability when harvested for flow cytometry. We first tested the tuft cell profile in *N. brasiliensis*-infected MIF-deficient mice, by staining sections for the Dclk1 (double cortin-like kinase-1) marker which within the intestinal epithelium is expressed only by tuft cells. Immunohistochemical staining indicated a striking deficiency in tuft cells in mice lacking MIF (Fig. 4a). Tuft cell numbers per crypt/villus axis (CVA) were then quantitated (Fig. 4b), confirming that the tuft cell hyperplasia developing in wild-type mice infected with *N. brasiliensis*, is abrogated in infected MIF-deficient mice. Specifically, Dclk1^+^ epithelial cells expanded 16-fold in the intestine of infected BALB/c mice, compared to a 2.5-fold increase in *Mif*^−/−^ mice. We then tested whether the 4-IPP inhibitor could recapitulate the effect of genetic ablation of MIF, and found that tuft cell numbers were greatly reduced in animals treated with 4-IPP, but intact in those receiving the DMSO vehicle alone, compared to untreated mice (Fig. 4c, d).

### Reduced goblet cell hyperplasia and RELMβ expression in MIF-deficient mice

We next examined goblet cell responses in the MIF-deficient mice, as these are also known to be highly elevated in intestinal helminth infection in an IL-13-dependent manner^51^. Small intestinal epithelial tissues of mice infected with *N. brasiliensis* for 6 days were stained with Periodic Acid-Schiff to enumerate goblet cells. Greater numbers of larger cells were evident in wild-type mice following infection, but no expansion was observed in the MIF-deficient hosts (Fig. 4e).

Sections were then stained for RELMβ protein, a goblet cell product with anti-helminth properties^52^. RELMβ was found to be intensely expressed in infected wild-type goblet cells but almost absent from MIF-deficient mice (Fig. 4f) with over a 100-fold fewer RELMβ^+^ epithelial cells in infected *Mif*^−/−^ animals (Fig. 4g). The corresponding level of *Retlnb* gene expression in the proximal small intestinal tissue was then assessed, and found to be 13-fold higher in wild-type than in MIF-deficient infected mice (Fig. 4h).

### MIF-deficient mice have abated Type 2 responses in the lamina propria and small intestine

**Fig. 5:**
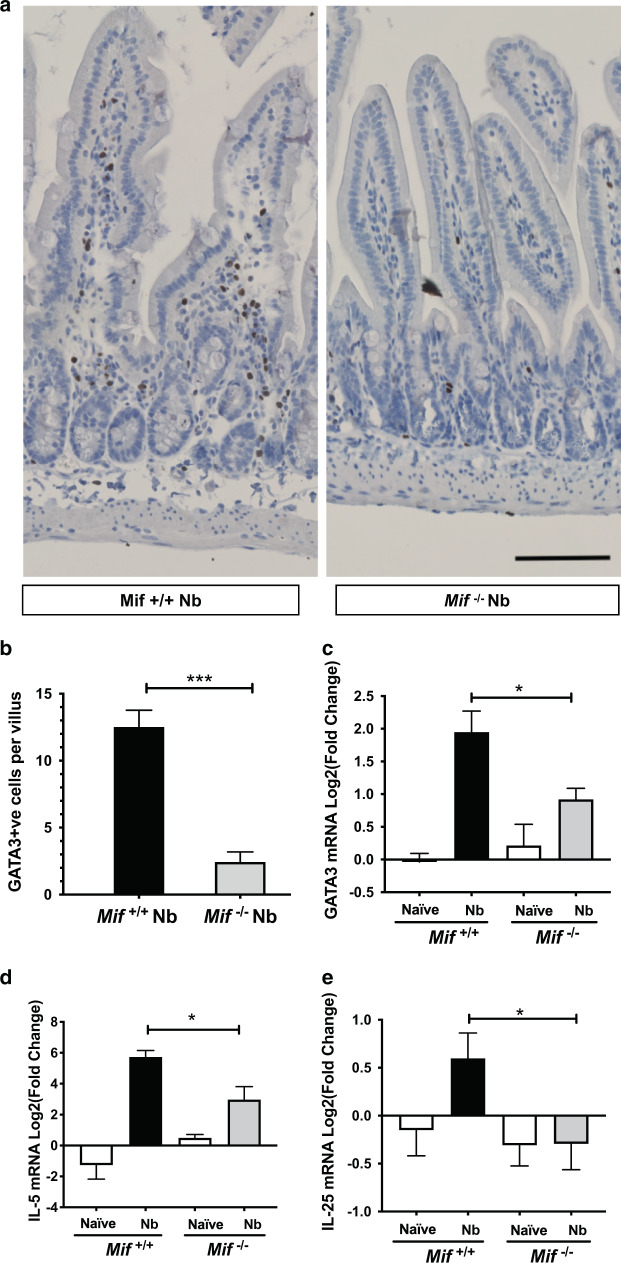
Type 2 immune cells and cytokines are reduced in the *Mif*^−/−^ small intestinal tissue during *N. brasiliensis* infection. *Mif*^+/+^ and *Mif*^−/−^ mice were infected with 400 L3 *N.brasiliensis* larvae by s.c. injection and small intestinal tissues taken for immunohistology of paraffin-embedded sections and RT-PCR evaluation of gene expression. **a** GATA3 staining of the lamina propria tissues from infected *Mif*^+/+^ and *Mif*^−/−^ mice. **b** Enumeration of GATA3^+^ cells in lamina propria of naive and infected *Mif*^+/+^ and *Mif*^−/−^ mice. **c** RT-PCR for *Gata3* mRNA in intestinal tissues from naive and infected mice. **d** RT-PCR for *Il5* mRNA in the same tissue samples. **e** RT-PCR for *Il17e* (IL-25) mRNA, as above. Data in **b** represent arithmetic means from one of two replicate experiments (*n* = 3 *Mif*^+/+^ and 4 *Mif*^−/−^ mice); data in **c**–**e** are geometric means and standard errors and are combined from two individually performed experiments (*n* = 7–8 animals); data were statistically analysed by unpaired *t* tests. *P < 0.05, ***P < 0.001.

In parallel, we stained sections for the transcription factor GATA3, which is upregulated in both ILC2s and Th2 cells, and found significantly reduced numbers of GATA3^+^ cells in the lamina propria of *N. brasiliensis*-infected MIF-deficient mice, ~20% of those present in wild-type animals) (Fig. 5a, b). While the immunohistochemistry did not discriminate between ILC2 and CD4^+^ Th2 populations, the parallel reductions shown by flow cytometry of MLN cells (Fig. 2) suggest that both compartments are compromised by the absence of MIF. Furthermore, we evaluated mRNA expression of *Gata3*, and *Il5* in harvested duodenal tissue from the two genotypes; in both cases (Fig. 5c, d), *Mif*^−/−^ mice showed dampened type 2 cellular activation. We also measured total IL-25 mRNA levels in the proximal small intestine, finding them to be significantly (2-fold) greater in *Mif* ^+/+^ mice than the levels transcribed in the MIF-deficient mice when infected with the nematode (Fig. 5e).

#### MIF is not required for in vitro differentiation of tuft cells in organoid cultures

The tuft cell deficiency in MIF-deficient mice could reflect an intrinsic defect in stem cell differentiation, or a role in the amplification circuit between tuft cells and ILC2s that results in tuft cell hyperplasia. To test whether MIF is influences differentiation at the stem cell level, we grew small intestinal organoids^53^ from wild-type and MIF-deficient cells. Addition of exogenous MIF did not induce a significant expansion of tuft cells in organoid cultures, but marked expansion of Dclk1^+^ intestinal tuft cells occurred cultures grown in the presence of IL4 and IL-13. Moreover, tuft cell differentiation was similar in organoid cultures grown from both wild-type and MIF-deficient intestinal stem cells, demonstrating that the developmental potential of precursor cells can be realised in the absence of MIF (Supplementary Fig. 4).

#### IL-25 rescues epithelial cell responses in the MIF-deficient mouse

**Fig. 6:**
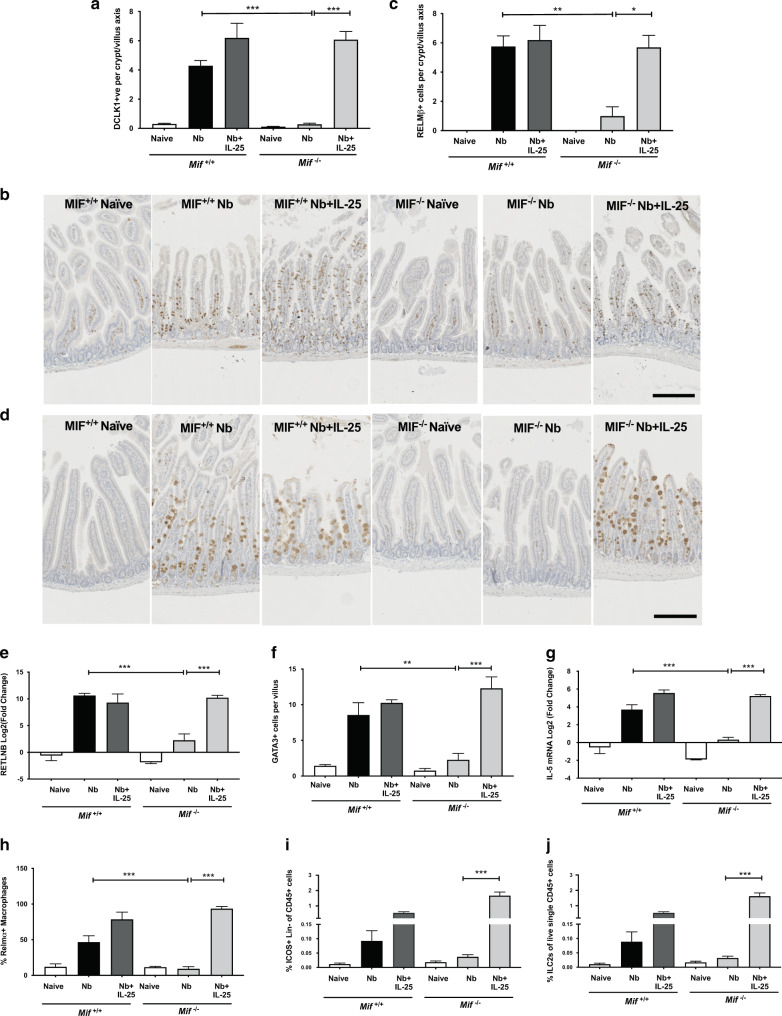
rIL-25 is able to rescue the epithelial cell phenotype in MIF-deficient mice. Recombinant IL-25 (800 ng in 200 μl PBS) or 200 µl PBS were administered i.p. to mice on days 1–5 following infection with *N. brasiliensis*. Tissues and cells were harvested on day 6 post-infection for subsequent analysis. **a** Enumeration of Dclk1-positive cells per small intestinal crypt-villus axis (CVA), based on a minimum of 100 crypt-villus units, from *Mif* ^+/+^ and *Mif* ^−/−^ mice as naive controls, *N. brasiliensis-*infected, and infected with treatment with rIL-25, all stained for Dclk1. **b** Representative images of intestinal tissue from *Mif* ^+/+^ and *Mif* ^−/−^ mice, from naive controls, *N. brasiliensis-*infected mice, and infected mice receiving rIL-25, all stained for Dclk1. **c** Enumeration of RELMβ^+^ cells per CVA in the same samples as **a**. **d** Representative images of RELMβ staining of intestinal tissue from MIF-deficient mice in similar sample sections as **b**. **e** Gene expression of *Retnlb* (RELMβ) assayed by RT-PCR on small intestinal tissue from infected mice (*n* = 6–7) and naive controls (*n* = 4). **f** Histological sections stained with an anti-GATA3 antibody and positive cells quantified per CVA on samples from infected mice (*n* = 3, except for Nb+IL-25, *n* = 2), and naive controls (*n* = 2–3). **g** Small intestinal tissue expression of *Il5* mRNA by RT-PCR. **h** The percentage of peritoneal macrophages (CD11b^+^F4/80^+^) expressing RELMα within the gate of live MLN CD45^+^ cells as determined by flow cytometry; *n* = 3 for all groups. **i** The percentage of ICOS^+^ Lin^−^CD4^−^ ILCs among live MLN CD45^+^ cells as determined by flow cytometry; from infected mice (*n* = 6–7) and naive controls (*n* = 4). **j** The percentage of GATA3^+^ ILCs among the same population as **i**. Data all represent two individually performed experiments, either shown as one representative result (**a**, **c**, **f**, **h**) or pooled data (**e**, **g**, **i**, **j**). Data analysed by one way ANOVA, and corrected by Sidak’s multiple comparison test. For all panels, **P < 0.01, ***P < 0.001.

Because tuft cells are the key source of IL-25, and as IL-25-deficient mice show compromised immunity to *N. brasiliensis*^48,54^, we explored whether exogenous IL-25 could reverse the epithelial cell defect of *Mif*^−/−^ mice. Parallel groups of wild-type and MIF-deficient mice received either rIL-25 or PBS i.p. daily from d1-5 of *N. brasiliensis* infection before harvesting tissues at day 6, and quantifying small intestinal Dclk1^+^ tuft cells. In infected wild-type mice, IL-25 administration caused a marginal increase (from 4.29 ± 0.36 to 6.19 ± 1.00 cells per CVA, *p* > 0.05). More dramatically, rIL-25 completely rescued the MIF^−/−^ phenotype, raising tuft cell densities from 0.28 ± 0.08 per CVA in control infected mice, to 6.08 ± 0.56 (*p* = 0.0005) in IL-25-treated MIF-deficient animals (Fig. 6a, b).

Similarly, IL-25 restored expression of RELMβ in infected *Mif*^−/−^ mice to wild-type levels, when quantified as RELMβ^+^ cells per CVA (Fig. 6c, d), and of the corresponding *Retnlb* mRNA transcript in duodenal tissue (Fig. 6e). In parallel, numbers of intestinal GATA3^+^ cells (Fig. 6f), and levels of duodenal *Il5* mRNA (Fig. 6g) were rescued by IL-25 administration, together with expression of the M2 marker RELM-α within the macrophage compartment (Fig. 6h). In addition, both MIF-sufficient and -deficient mice showed expansion of ILC2 populations when given IL-25, although *N. brasiliensis* induced a response only in the wild-type animals (Fig. 6i, j).

#### Expression of MIF and MIF receptors during helminth infection

MIF is produced by multiple cell types in the steady-state, including intestinal epithelial cells^32,55^; following *H. polygyrus* infection, MIF expression rises in key cell types such as tuft cells^56^. To investigate whether immune system cells also produce MIF during *N. brasiliensis* infection, and as recovery of viable lamina propria cells from helminth-infected mice is problematic, we purified lymphoid and myeloid populations from the peritoneal cavity of uninfected and infected mice, and measured *Mif* mRNA levels by RT-PCR. As shown in Fig. S5, macrophages and eosinophils showed high levels of *Mif* expression following infection, while both B and T lymphocytes remained low, and did not significantly change on infection.

MIF is also known to signal via the extracellular signal-regulated kinases and mitogen-activated protein kinases^57^, activated through a combination of non-canonical receptors, namely CXCR2 and CXCR4^58^, as well as CXCR7/ACKR3^59,60^, together with CD74^61^ as a co-receptor^10^. To identify cell populations which may be responsible for driving the effects of MIF on cellular recruitment, we assayed expression of these receptors by flow cytometry. MLN and peritoneal cells were harvested from wild-type mice infected with *N. brasiliensis*, and naive controls, co-staining to differentiate CD11b^+^F4/80^+^ macrophages, CD4^+^GATA3^+^ Th2 cells, CD11b^+^ SiglecF^+^ eosinophils (in the peritoneum) and Lin^−^CD4^−^ICOS^+^ ILCs including GATA3^+^ ILC2s (in the MLN).

**Fig. 7:**
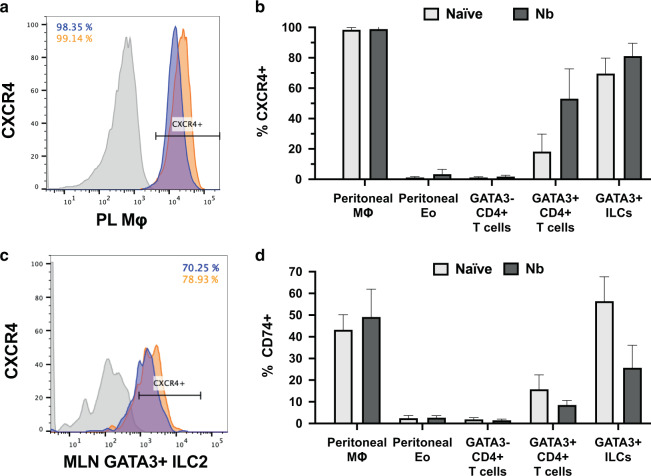
MLN and peritoneal lavage cell receptor expression at D6 of *N.brasiliensis* infection. Expression of chemokine receptors associated with MIF responsiveness, measured on immune cells, from peritoneal cavity and mesenteric lymph nodes of naive and infected BALB/c mice. Expression of CXCR4 in peritoneal macrophages (**a**) and eosinophils, and MLN T cells and ILCs (**b**, **c**) from naive (purple, grey) and *N. brasiliensis-*infected (orange, black) BALB/c mice. **d** Expression of CD74 in the same populations in BALB/c mice. Data all represent two individually performed experiments, which were pooled for **b** and **d**.

No significant expression of CXCR2 was found in these populations, but 100% of peritoneal macrophages were CXCR4-positive (Fig. 7a). Notably, ILC2 and Th2 cells showed significant baseline CXCR4 expression, enhanced following helminth infection, reaching up to 80% of ILC2s (Fig. 7b, c). Expression of the CD74 co-receptor was found on ~50% of both ILC2s and peritoneal macrophages, and although downregulated by infection in the former population (Fig. 7d), many ILC2s expressed the combination of CXCR4 and CD74. Finally, ACKR3 expression, measured in a GFP-reporter strain, was found only on a minority of peritoneal macrophages, and on no MLN population (Fig. S6). In further studies, we examined whether ILCs could be directly activated by MIF, but were not able to significantly stimulate cytokine production or proliferation of ILCs by MIF, either by in vitro stimulation of purified ILC2s (Fig. S7a), or administration in vivo even in the presence of exogenous IL-25 (Fig. S7B), but activation may be context-dependent requiring other tissue factors yet to be identified.

## Discussion

Although MIF was among the first cytokines to be discovered, many key aspects of its biology have remained enigmatic^7,10,62–64^. While it is a major pro-inflammatory mediator in settings such as bacterial infection and sepsis, acting through macrophages^10^, the effects of MIF on other cell populations, and its general role in other contexts such as type 2 immunity, are less well understood. In this report, we now define the MIF-dependent populations in type 2 innate immunity, including ILC2s, eosinophils, tuft and goblet cells, as well as M2 macrophages stimulated by helminth infection. These findings confirm earlier work that the effects of MIF are highly context-dependent, and suggest that its role is not as an instructive or directional cytokine, but as a co-activator or checkpoint for innate immunity in its different modes. Specifically within the setting of type 2 immunity, we can now suggest that MIF acts to activate ILC2s as well as M2 macrophages, either directly or through an intermediary cell type. In either case, activation of ILC2s is necessary to promote eosinophilia, tuft cell expansion, and goblet cell expression of RELMβ. Our findings concord with earlier studies reporting loss of eosinophilia in the absence of MIF, in settings both of helminth infection^28^ and allergy^27,65^, confirming that MIF is required for type 2 innate immunity.

Our report of compromised type 2 immunity to *N. brasiliensis* in MIF-deficient mice follows our previous work showing similar susceptibility of this construct to another intestinal helminth, *H. polygyrus*^32^. These two parasite species have quite distinct properties, with *H. polygyrus* being primarily susceptible to type 2 myeloid cell effectors, particularly M2 macrophages, while actively suppressing the tuft cell and goblet cell differentiation characteristic of *N. brasiliensis* infection^66^. *N. brasiliensis* also differs in migrating through the lung; however the similar larval recoveries in both wild-type and MIF^−/−^ mice indicate that, in this model at least, MIF deficiency results in a failure of intestinal immunity that is dramatically evident in the loss of critical tuft cell and goblet cell responses.

In our previous study we were able to show that M2 differentiation was delayed in MIF^−/−^ mice alongside deficits in ILC2 and eosinophil responses, and that rMIF could rescue and promote M2 macrophage activation both in vitro and in vivo. Our current report, therefore, generalises this finding to show that two populations essential for immunity to *N. brasiliensis*, ILC2s and tuft cells, also require the presence of MIF for their expansion.

IL-25 is released from intestinal tuft cells stimulated by helminth infection, and acts on ILC2 cells to release type 2 cytokines, including IL-5 and IL-13^43^. These mediators drive the response of eosinophils, M2 macrophages and CD4^+^ Th2 cells, with IL-13 acting on stem cells to promote hyperplasia of the tuft cell compartment, creating a feed-forward amplification loop^40–42,44,45^. This amplification fails to occur in the absence of MIF. As tuft cells can develop in MIF-deficient organoid cultures, and the effects of MIF deficiency impact each of the type 2 cell populations, we surmised that MIF^−/−^ phenotype affects the IL-25-responding population of ILC2s. Correspondingly, we were able to rescue the phenotype of MIF-deficient mice with exogenous IL-25. In addition, in a separate study we noted that the potent allergen *Alternaria alternata* can evoke a strong IL-33 response in the airways of MIF-deficient mice, while exogenous IL-33 can induce similar ILC2 activation compared to wild-type animals (K.J.F, McSorley, H.J. and R.M.M., unpublished observations). These results demonstrate that there is not an absolute requirement for MIF, but that it may be required primarily at low alarmin doses, particularly at the initiation and expansion phases of the response.

Mechanistically, MIF is thought to act through a receptor complex of CD74 (a fragment of MHC Class II invariant chain) with the chemokine receptors CXCR2, CXCR4 and CXCR7 or ACKR3^58^, with CD44 as a signalling component^67^. We have more recently established that tuft cell responses to *N. brasiliensis* are intact in CXCR2-deficient mice (S.L., F.V., Dyer, D., Graham, G., and R.M.M., in preparation), suggesting that one of the other receptor subunits is critical in helminth infection. In this context, we are intrigued by our finding that CXCR4, and not CXCR2, is expressed on ILC2 cells in vivo, consistent with earlier reports profiling gene expression in various ILC subsets^68^, and direct analysis of gastric^69^ and IL-25-induced ILC2s in mice^70^. As we did not find direct activation of ILCs by MIF, it may be that other factors yet to be identified, or combinations of stimuli we did not examine, are required. Studies are now underway to establish if MIF directly ligates CXCR4 on ILCs, and if the in vivo effects of MIF can be abrogated with CXCR4 antagonists. Interestingly, a previous study of *Schistosoma mansoni* infection reported significantly reduced IL-5 and eosinophilia in mice given the antagonist AMD3465^71^. Since we do not find expression of MIF-associated chemokine receptors on eosinophils, it appears possible that our results, and earlier work demonstrating abated eosinophilia in the absence of MIF^27,28,65^, reflects an indirect induction of eosinophils by another, MIF-responsive, CXCR4^+^ cell type, most likely the ILC2 subset.

Interactions have also been reported between MIF and CXCR7 (also known as atypical chemokine receptor-3, ACKR3)^59,60^. Little is known of these receptors in the setting of helminth infection, but ACKR3 expression in wild mice (*Apodemus sylvaticus*) is one of 4 loci to correlate most negatively with burdens of *H. polygyrus*^72^, while its ligand CXCL12 (which is the canonical ligand for CXCR4) is required for immunity to a tissue-dwelling nematode *Litomosoides sigmondontis*^73^. Further studies are necessary to unravel the role of both canonical and atypical chemokines in immunity to intestinal parasites, as well as the involvement of the corresponding receptor molecules.

The failure of MIF-deficient, or 4-IPP inhibitor-treated, mice to promptly expel *N. brasiliensis*, contrasts with an earlier report from another laboratory that found stronger type 2 immunity in the absence of MIF^74^. In this report, both MIF-sufficient and -deficient mice expel the parasite, although the deficient mice do so more rapidly, and the phenotype of greater resistance was replicated with a pharmacological inhibitor, sulforaphane, with a similar mode of action blocking tautomerase activity, as the 4-IPP we employed in our experiments. The disparity between studies on the same gene-targeted mouse line^75^ is not readily explicable, as we verified the susceptibility phenotype reported here with two independent strains of the parasite (Fig. 1a–c), and in two different animal facilities. However, we do note that the greater resistance reported by Damle and colleagues was attributed to enhanced CD4^+^ Th2 activity in MIF-deficient mice, evaluated for example by transfer of wild-type and *Mif*^−/−^ T cells into RAG recipients prior to *N. brasiliensis* infection, and that these authors did not measure innate immune parameters other than goblet cell hyperplasia. It is possible that in a different microbiological environment, stronger IFNγ responses promoted by MIF^76^, or elevated IL-17A^74^ may act to delay Th2 immunity in MIF-sufficient, but not MIF-deficient mice.

In conclusion, the ‘multi-tasking’ atypical chemokine MIF has been shown to dramatically impact on key type 2 immune responses during helminth infection, and to do so across a range of cell types from eosinophils, M2 macrophages, goblet cells and, most strikingly, on tuft cells. Future work will aim to delineate key MIF-mediated pathways, and whether these involve co-stimulation of ILCs, or act through macrophages in a novel MIF-dependent mechanism. Our data also reiterate the central role of IL-25 in activating protective immunity to helminths as demonstrated in multiple settings^48,54,77–81^. MIF is therefore an important physiological player in intestinal immunity that should be incorporated into our new understanding of how the complex protective response to parasites is stimulated and orchestrated.

## Materials and methods

### Mice and parasites

BALB/c and MIF-deficient mice on the BALB/c background were bred in-house and housed in individually-ventilated cages (IVCs) according to UK Home Office guidelines. The *Mif*^−/−^ construct contains a neomycin cassette disrupting exons 2 and 3 of the *Mif* gene^75^. ACKR3-GFP mice (Ackr3^tm1Litt^)^82^ were kindly provided by Prof. Gerard Graham, University of Glasgow, UK.

Infections employed *N. brasiliensis* maintained as previously described^83^. NES was collected as spent culture medium collected over 7 days from adult *N brasiliensis* as described^84^, centrifuged at 400 *g* for 10 min to remove eggs, and diafiltrated into PBS over a 3000 MW cut-off membrane in an Amicon, sterilized by passage through a 0.2 μm filter (Millipore) and frozen at −80 °C.

Adult worm counts were conducted after small intestines were removed and sliced longitudinally. Egg counts were performed on 3–4 fecal pellets which were weighed and resuspended in 2 ml dH_2_O; 2 ml of saturated salt solution (400 g NaCl in 1 L dH_2_O) was then added and eggs enumerated using a McMaster egg counting chamber. Egg counts are represented as eggs/g fecal material.

### In vivo administrations

Inhibition of MIF activity in vivo was performed by administration of 1 mg of MIF inhibitor, 4-IPP (Tocris Bioscience Cat. No.3249)^50^ dissolved in DMSO, or DMSO alone, intraperitoneally in 50 μl every other day, during *N brasiliensis* infection (adapted from ref. ^26^). Administration of cytokines to mice involved 400 or 800 ng recombinant IL-13 or IL-25, and/or 1 µg MIF i.p. on each of days 1–5 following infection, with analysis of tissues on day 6 post-infection.

### Cell isolation and culture

MLN cell suspensions were prepared directly by passage through 70 μm nylon filters (BD) and placed in RPMI1640 (Gibco) supplemented with 10% FCS, 1% PenStrep (Gibco) and 1% L-glutamine (Gibco). Cells were restimulated for 72 h at 37 °C with either media alone or NES at a final concentration of 1 μg/ml with 1 × 10^6^ cells, in triplicate. Peritoneal exudate cells were collected by washing the peritoneal cavity with 2 × 5 ml ice-cold RPMI1640. Red blood cells (RBC) were removed by adding 3 ml RBC lysis buffer (Sigma) for 4 min, and washing with supplemented RPMI. Peritoneal lavage used for ELISA analysis consisted of the first 5 ml wash taken from the centrifuged sample to remove cell debris. Bronchoalveolar lavage was collected for ELISAs by washing the lungs with 1 ml ice-cold PBS. Lung tissue was digested in HBSS (Gibco) supplemented with 4 U/ml Liberase TL (Roche) and 160 U/ml DNAse 1 (Sigma). Tissue was incubated at 37 °C for 25 min, passaged through 70 μm nylon filters (BD) and RBC-lysed before cells were used for flow cytometric analysis.

### Flow cytometry

Cells were stained in 96-well round-bottomed plates. Prior to antibody staining, cells were washed in PBS and stained with eBioscience Fixable Viability Dye eF506 (Invitrogen) at a 1/1000 dilution in 100 μl PBS for 20 min at 4 °C. Then, Fc receptors were blocked in 50 μl of FACS buffer containing 1 μg/10^6^ cells of anti-CD16/CD32 (Invitrogen) for 20 min at 4 °C. Samples were then surface stained for 20 min in 20 μl of FACS buffer containing a combination of the following antibodies:

**Table:** 

Anti-mouse lineage cocktail (BioLegend)	Anti-mouse CD3, clone 17A2 Anti-mouse Ly-6G/Ly-6C, clone RB6-8C5; Anti-mouse CD11b, clone M1/70; Anti-mouse CD45R/B220, clone RA3-6B2 Anti-mouse TER-119/Erythroid cells, clone Ter-119
Anti-human/mouse ICOS (BioLegend)	Clone C398.4A
Anti-mouse CD3 (BioLegend)	Clone 17A2
Anti-mouse CD4 (BioLegend)	Clone RM4–5
Anti-mouse CD11c (BioLegend)	Clone N418
Anti-mouse CD11b (BioLegend)	Clone M1/70
Anti-mouse F4/80 (BioLegend)	Clone BM8
Anti-mouse Siglec-F (BD)	Clone E50-2440 (PE and BUV395)
Anti-mouse Gr1 (BioLegend)	Clone RB6-8C5
Anti-mouse CXCR2 (BioLegend)	Clone SA044G4 (AF647)
Anti-mouse CXCR4 (BioLegend)	Clone L276F12 (BV711)
Anti-mouse CD45 (BioLegend)	Clone 30-F11
Anti-mouse CD74 (BD)	Clone In-1 (BUV395)

To measure intracellular IL-5 and IL-13 expression, cells were first stimulated for 4 h at 37 °C in the presence of PMA (50 ng/ml), Ionomycin (1 μg/ml), and Brefeldin A (10 μg/ml) (all from Sigma). To detect intracellular antigens, cells were fixed for 30 min at 4 °C in eBioscience IC Fixation Buffer (Invitrogen), permeabilized by washing twice in 200 μl of eBioscience Permeabilization Buffer (Invitrogen) and then stained for intracellular cytokine expression in Permeabilization Buffer using the following antibodies:

**Table:** 

Anti-mouse IL-5 (Invitrogen)	Clone TRFK5
Anti-human/mouse Arginase-1 (R&D)	Clone IC5868P
Anti-mouse RELM-α (Peprotech)	Cat. No. 500-P214
Zenon Alexa Fluor-647 Rabbit IgG Labeling Kit (Invitrogen)	Cat. No. Z25308
Anti-mouse YM1/Chitinase 3-like 3 Biotinylated Antibody (R&D)	Cat. No. BAF2446

To label transcription factors, samples were stained for surface markers after which cells were permeabilized for 1 h at 4 °C in Fix/Perm solution (eBioscience Foxp3 staining set), and then washed twice in 200 μl of Perm/Wash (eBioscience Foxp3 staining set) and stained with anti-mouse GATA3 (clone L50-823, BD) in Perm/Wash solution. Thereafter, cells were washed twice in 200 μl of FACS buffer before acquisition on the LSR II, Celesta or Fortessa flow cytometers (BD Bioscience) and subsequently analyzed using FlowJo (BD).

### Cytokine ELISAs

Cytokine levels were detected in culture supernatants and BAL fluid by ELISA using monoclonal capture and biotinylated detection antibody pairs as follows, used at concentrations optimised previously: IL-4 (11B11 + BVD6-24G2 (BD Pharmingen)); IL-5 (TRFK5 + TRFK4 (eBioscience)); IL-13 (eBio13A + eBio1316H (eBioscience). *p*-nitrophenyl phosphate (pNPP, 1 mg/ml, Sigma) was used as a substrate. OD was measured at 405 nm on a Precision microplate reader (Molecular Devices) and data analysed using Softmax Pro software.

### Gut homogenate

Approx. 1 cm small intestine was homogenised in 500 μl 1× lysis buffer (Cell Signalling Technology Inc) plus 5 μl phenylmethanesulfonylfluoride solution (Sigma) using a TissueLyser (Qiagen). Samples were centrifuged at 10,000 *g* for 10 min to remove debris and supernatants added to ELISAs, at a 1:10 dilution, to measure RELM-α (Peprotech) and Chil3 (R&D). Levels were normalised to total protein content measured using a Bradford assay. The same ELISA sets were used to analyze PL levels of RELM-α and Chil3.

### Immunohistochemistry

Immunohistochemistry on intestinal sections was performed as previously described^40^. Transverse sections were made from 2 cm of paraffin-embedded small intestine, at a thickness of 4 μm using a microtome. Sections were deparaffinized by immersing slides in several baths of xylene, and then hydrated through 100%, 95 and 70% ethanol successively. Antigen retrieval was undertaken with citrate buffer (20 mM citric acid, 0.05% Tween 20 at pH 6.4) warmed to 95 °C for 20 min. Sections were blocked in 1x TBS with 1% BSA, 2% normal horse serum, 0.1% Triton X-100 and 0.05% Tween 20 for 30 min at room temperature and then incubated with rabbit anti-mouse Dclk-1 (Abcam 31704), rabbit anti-mouse RETNLB (Antibodies Online ABIN465494) or rabbit anti-mouse GATA3 (Abcam EPR16651) at 1:2000 dilution in block buffer, and left overnight at 4 °C. ImmPRESS polymer kits containing peroxidase-conjugated anti-rabbit (Vector Laboratories MP-7402) or anti-mouse (MP-7401) antibodies were added and slides left for 30 min at room temperature. Slides were washed twice in PBS and DAB peroxidase solution (Sigma) was added for 5 min (until a brown stain had developed). With water washes in between, slides were counterstained with Harris hemotoxylin (Sigma), and dehydrated through 75%, 95 and 100% ethanol and xylene baths before permanent mounting. Pictures were taken using a slide scanner (Nanozoomer, Hamamatsu).

### RNA extraction and quantitative PCR

RT-PCR was performed using the gene-specific primer pairs given in Table S1. Approx. 0.5 cm of the uppermost part of the duodenum was placed into 1 ml of TrIzol (Invitrogen) for 2 min, and then extracted in a TissueLyser (Qiagen) according to the manufacturer’s protocol at a frequency of 25 Hz. mRNA isolation was performed with the Qiagen RNeasy kit (Qiagen 74106) as previously described^32^ in which 140 μl of chloroform was added to each sample, before vigorously shaking for 15 s and standing at room temperature for 5 min. Samples were then centrifuged for 15 min at 12,000 *g* at 4 °C. The upper aqueous phase was transferred to a new collection tube without interrupting the white phase between the two layers. 1.5 volumes of 100% ethanol were added, mixing thoroughly. Up to 700 µl was placed in RNeasy spin columns and centrifuged at 8000 *g* for 1 min at room temperature. 350 µl of Buffer RW1 (RWT for the microRNA kit) was added and centrifuged at 8000 *g* for 1 min. 10 µl of DNase1 was added to 70 µl of RDD buffer. 80 µl was placed onto spin columns and incubated for 15 min. Purified mRNA was washed once in 70% ethanol, and allowed to air dry before being dissolved in 50 μl of DEPC-treated water; 15 μl RNA was treated with DNAse (DNAFree kit, Ambion), concentrations were determined using a Nanodrop 2000 (Thermo Scientific), and samples reverse-transcribed using 1–2 μg of RNA with M-MLV reverse transcriptase (Promega). A PCR block (Peltier Thermal Cycler, MJ Research) was used for the transcription reaction at 37 °C for 60 min.

Gene transcript levels were measured by real-time PCR cDNA was diluted 1/5 and a 1 µl of each sample pooled for use as a top standard. Standards were serially diluted ¼ to obtain at least 6 standards. 2 µl of standard was added to 4 µl of maser mix and added to a 384 well plate. qPCR runs were performed using the Applied Bioscience Quantstudioflex 7. Analysis was performed by a delta-delta Ct method. Relative gene expression levels were calculated using either values for the same gene in naive tissues, or GAPDH expression in the same mRNA samples.

### Organoid culture

Organoids were cultured from crypts isolated from the proximal 10 cm of small intestine (duodenum) from 8 to 12 week old BALB/c and MIF-deficient mice. Briefly, the duodenum was cut into 2 mm pieces and washed 3 times with cold PBS before incubation with 2 mM EDTA in PBS for 30 min at 4 °C. After removal of EDTA solution, cold PBS was added and crypts isolated from basal membrane by pipetting. This procedure was repeated with more vigorous pipetting to create 6 fractions. Fractions with enriched crypts were identified using microscopy and pooled through a 70 μm cell strainer (Greiner) and crypts were then pelleted at 300 *g* for 3 min at 4 °C. Crypts were resuspended and centrifuged at 100 *g* for 3 min at 4 °C to separate from single cells. The pellet was then resuspended in 10 ml Basic Medium, composed of Advanced DMEM/F12 (Gibco) supplemented with 1% PenStrep, 1% L-glutamine and 10 mM HEPES (all Gibco). Crypt numbers were counted by microscopy, and 500 crypts in 40 μl Matrigel (Corning) were seeded into each well of a 24-well flat-bottomed plate (Corning). After incubation for 10 min at 37 °C, wells received 400 μl of complete crypt medium, composed of Basic Medium plus 50 ng/mL murine EGF (Invitrogen), 100 ng/mL murine Noggin (Peprotech) and 500 ng/ml murine R-spondin-1 (R&D). For initial plating only, 3 μM CHIR99021 (Miltenyi) was added. Crypts were then cultured at 37 °C in 5% CO_2_, medium changed every 2–3 d and organoids passaged once a week by dissociation and washing in cold Basic Medium, re-seeding at 500 crypts per well in 40 μl Matrigel. Organoids were passaged at least 3 times and seeded out in 4- or 8-well chamber slides (Thermo Scientific) before use in stimulations and microscopy. Organoids were stimulated with IL-4 (Miltenyi) and IL-13 (Peprotech) at 400 ng/mL and murine MIF (R&D) at 10 μg/mL for 48 h before fixation and staining.

### Organoid immunofluorescence staining

For immunofluorescence staining, cultured organoids were washed in PBS twice and then fixed in 4% paraformaldehyde for 20 min at room temperature. Organoids were then permeabilized with PBS containing 0.5% Triton X-100 for 10 min at 4 °C, followed by rinsing 3 times with PBS containing 100 mM glycine. Organoids were blocked with IF buffer (PBS containing 0.1% BSA, 0.2% Triton X-100, 0.05% Tween-20 and 10% FCS) for 1 h at room temperature before incubation with 1/1000 anti-mouse Dclk1 (Abcam) in antibody diluent (Invitrogen) at 4 °C overnight. After rinsing 3 times with IF buffer, slides were incubated with anti-rabbit FITC (Dako) for 1 h at room temperature. Slides were then washed with IF buffer followed by 3 rinses with PBS before being mounted with Vectashield mounting media containing DAPI (Vector Laboratories). Slides were imaged using DeltaVision Core microscope and softWoRx software (GE Healthcare). Resulting image files were analysed using ImageJ/Fiji^85^.

### Software and statistics

All statistical analyses were performed using Prism 7 (Graphpad Software Inc.). Error bars on graphs display mean and standard errors of the mean (SEM). For comparisons of two groups Student’s *t* test was used, and for multiple comparisons, ANOVA including Sidak’s correction; in some cases nonparametric statistics were applied. n.s. = not significant, *P < 0.05, **P < 0.01, ***P < 0.001.

## Supplementary information

Supplementary Material

## Data Availability

All data needed to evaluate the conclusions of the paper are present in the manuscript or the Supplementary Materials.
