# Supplementary material for: The IL-25-dependent tuft cell circuit driven by intestinal helminths requires macrophage migration inhibitory factor (MIF)
Source: Mucosal Immunol. 2022 Mar 14;15(6):1243–56. doi: 10.1038/s41385-022-00496-w (PMC9705247; doi:10.1038/s41385-022-00496-w)
Supplement: Supplementary file 1 — Supplementary Material [file 41385_2022_496_MOESM1_ESM.pdf]

The IL-25-dependent tuft cell circuit driven by intestinal helminths requires macrophage migration inhibitory factor (MIF)

Fumi Varyani[1,2], Stephan Löser[1], Kara J. Filbey[2,3], Yvonne Harcus[2], Claire Drurey[1], Marta Campillo Poveda[1], Orhan Rasid[1], Madeleine P.J. White[1], Danielle J Smyth[1], François Gerbe[4], Philippe Jay[4] and Rick M Maizels[1*]

Supplementary Material

Fig. S1. Type 2 cytokines in peritoneal lavage of *N. brasiliensis* infected MIF$^{+/+}$ and MIF$^{-/-}$ mice.

Fig. S2. MIF deficiency leads to impaired eosinophil recruitment and alternative macrophage polarization in the airways.

Fig. S3. Flow cytometry gating strategy for ILCs and macrophages

Fig. S4. Tuft cell differentiation in organoids of MIF-deficient intestinal cells

Fig. S5. MIF gene expression is significantly upregulated in macrophages and eosinophils following *N. brasiliensis* infection.

Fig. S6. Peritoneal lavage and MLN cell ACKR3 (CXCR7) expression in *N.brasiliensis* infection.

Fig. S7. MIF does not directly activate ILCs *in vitro* or *in vivo*

Table S1. Primer sequences for RT-PCR of gene expression

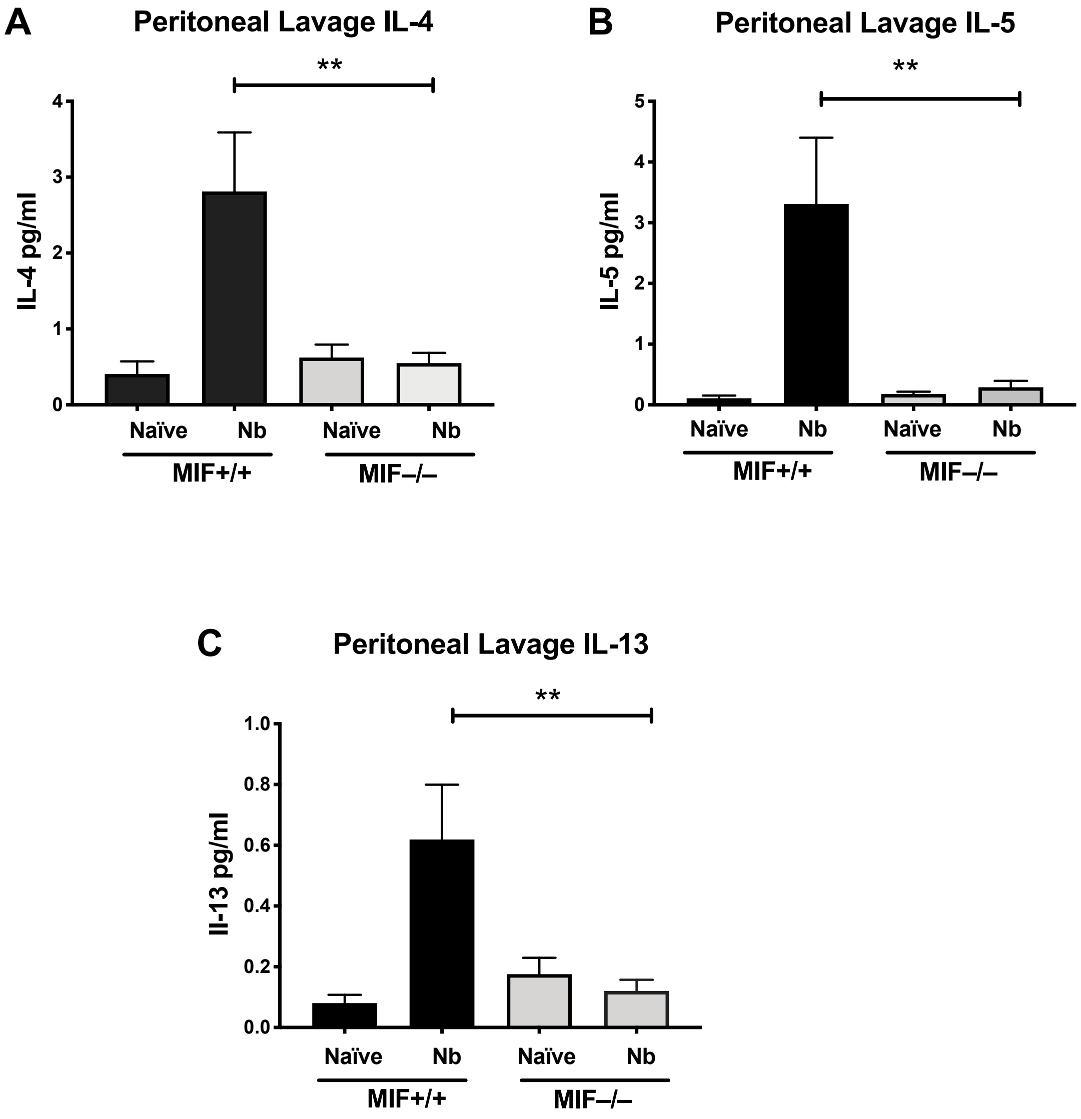

Supplementary Figure 1.

Type 2 Cytokines in Peritoneal Lavage of *N. brasiliensis* infected MIF$^{+/+}$ and MIF$^{-/-}$ mice.

MIF$^{+/+}$ and MIF$^{-/-}$ mice were infected with *N. brasiliensis* L3 larvae s.c. and sacrificed at d6 p.i.. Peritoneal lavage fluid was taken to quantify cytokine concentrationss of IL-4 **(A)**, IL-5 **(B)** and IL-13 **(C)** by cytometric bead array (CBA). Data presented are pooled from two independent experiments. Data are arithemetic means and standard errors, analysed by unpaired t tests. ** p<0.01.

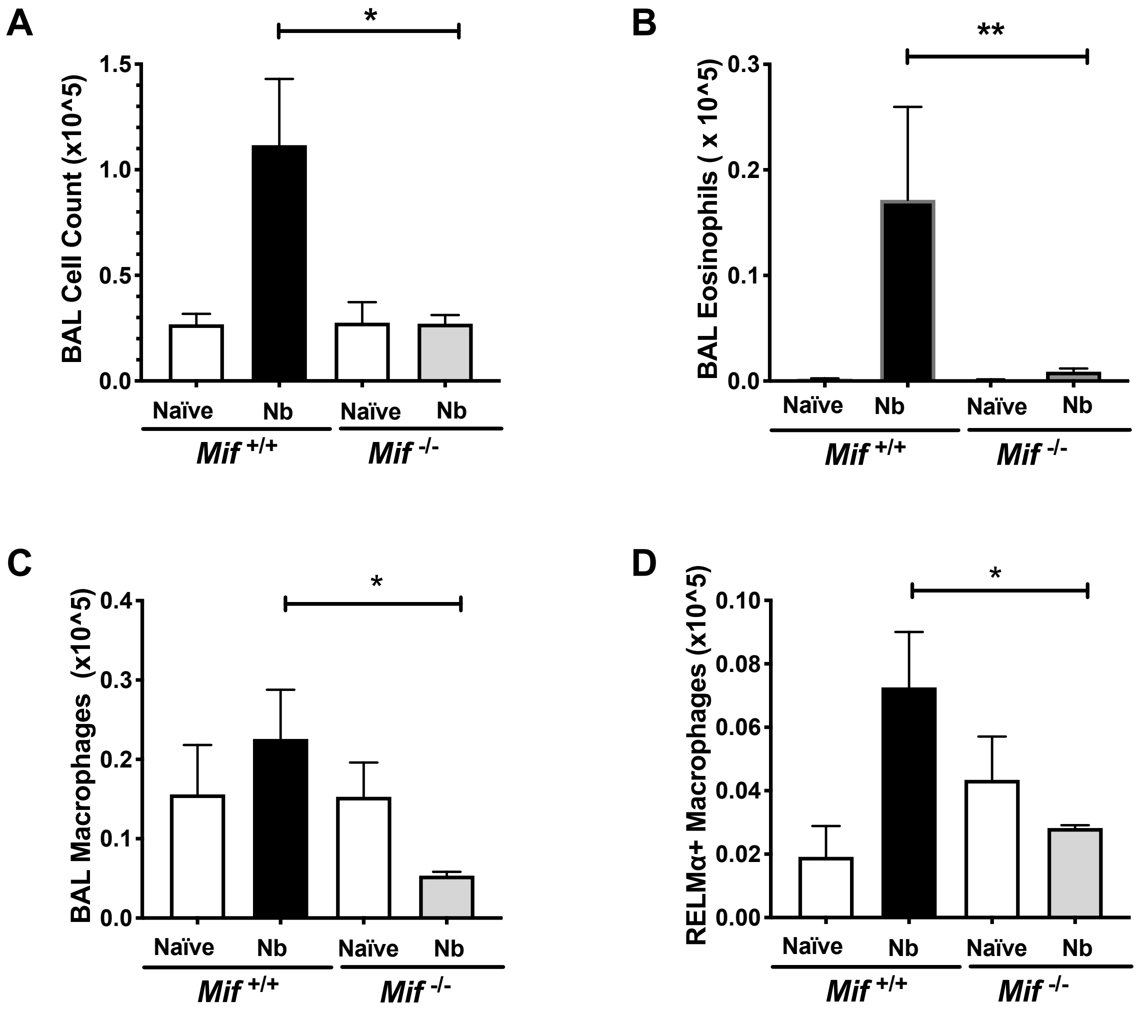

Supplementary Figure 2.

MIF deficiency leads to impaired eosinophil recruitment and alternative macrophage polarization in the airways.

$MIF^{+/+}$ and $MIF^{-/-}$ mice were infected with 400 L3 *N. brasiliensis* larvae by s.c. injection, and samples recovered by bronchoalveolar lavage (BAL) at day 3 of infection.

(A) Total cells in BAL fluid.

(**B**) Siglec F^{+} eosinophils, (**C**) macrophages and (**D**) RELMα+ alternatively activated macrophages in BAL identified by flow cytometry.

Data are presented as arithmetic means and standard errors, and represent one experiment; data were statistically analysed by unpaired t tests. * $p<0.05$, ** $p<0.01$.

Varyani *et al.* Supplementary Figure 3

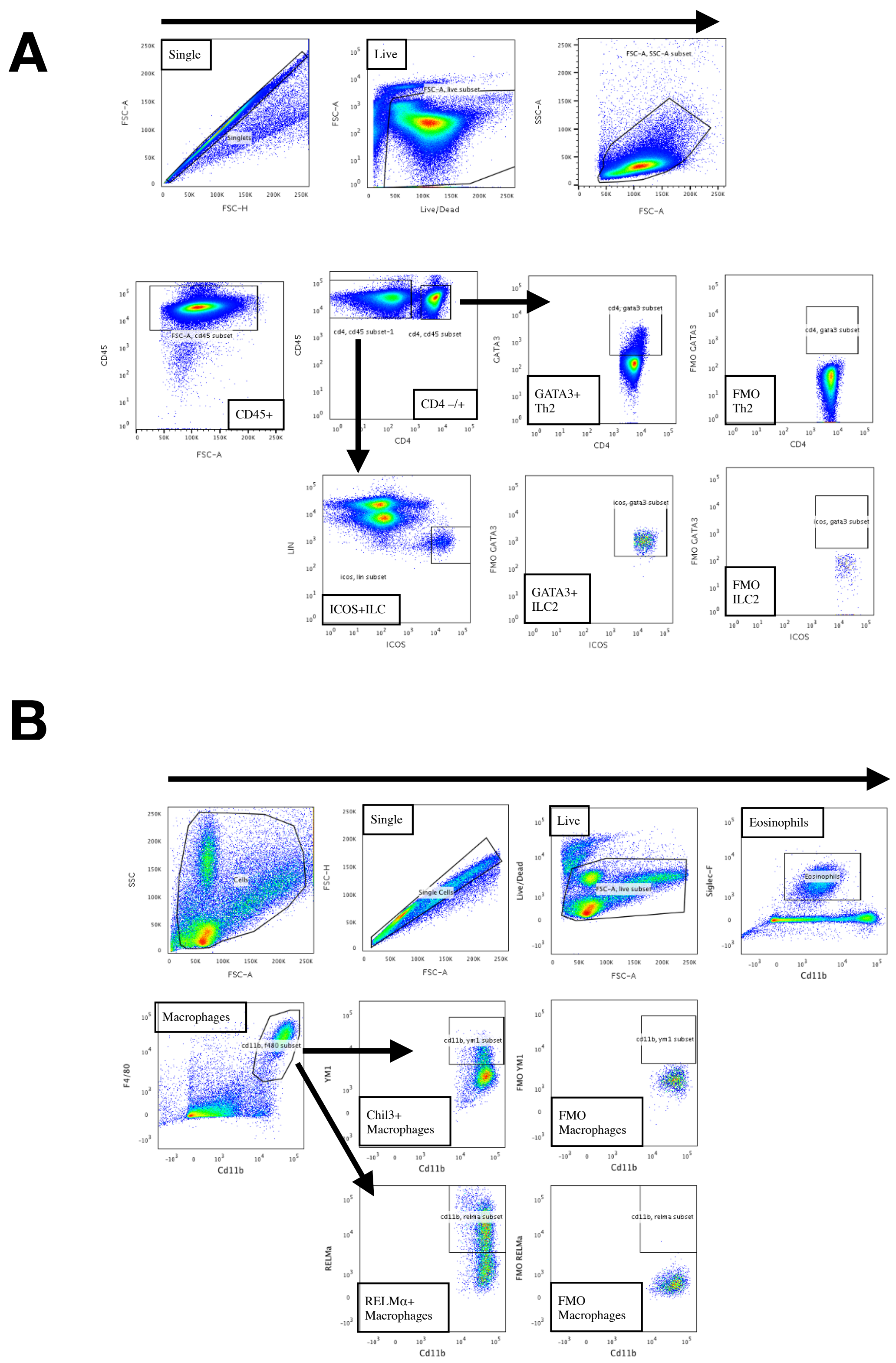

Supplementary Figure 3.

Flow cytometry gating strategy for ILCs and macrophages.

Gating strategies for mesenteric lymph node (MLN) cells (A) and peritoneal cavity cells (B) showing first selection of single live cells, then staining CD45, CD4, ICOS and GATA3 (A), and CD11b, F4/80, Siglec F, Chil3/Ym1 and RELMα (B).

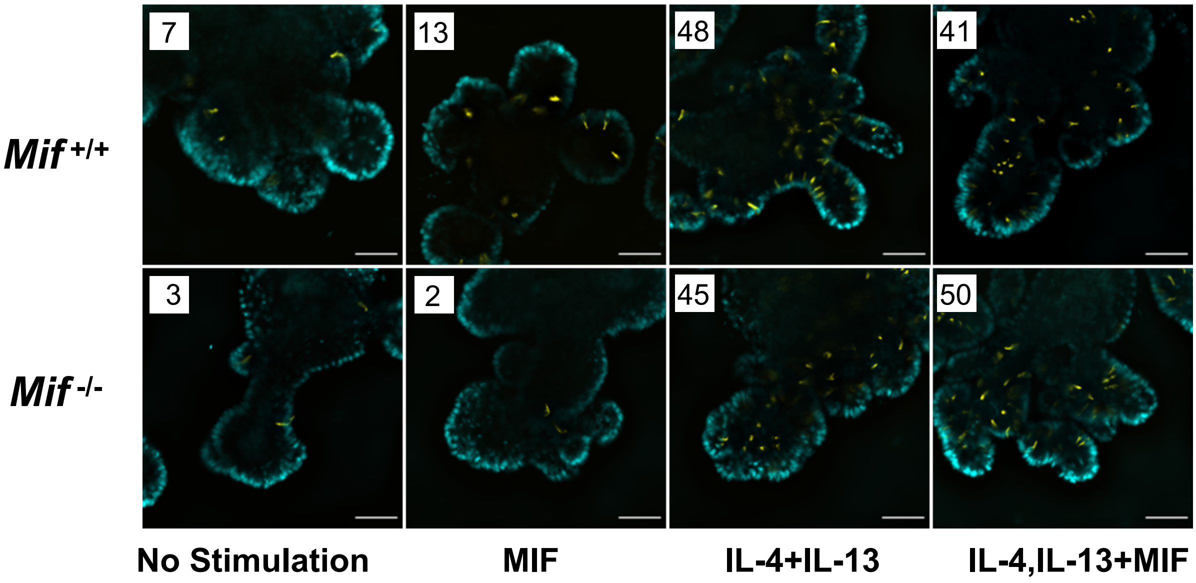

Supplementary Figure 4

MIF is not required for tuft cell differentiation in organoid cultures.

Tuft cell staining in small intestinal organoids, following incubation with: Media (no stimulation); MIF (10 µg/ml); IL4 and IL13 (both at 400 ng/ml); or IL4 and IL13 (both 400 ng/ml) + MIF (10 µg/ml). Following fixation, organoids were stained with anti-Dclk1 to label tuft cells; total tuft cell numbers in each organoid are shown in inset boxes. The fluorescence micrographs illustrate tuft cells in green (Dclk1+), and nuclei in cyan (DAPI); scale bars are 50 µm.

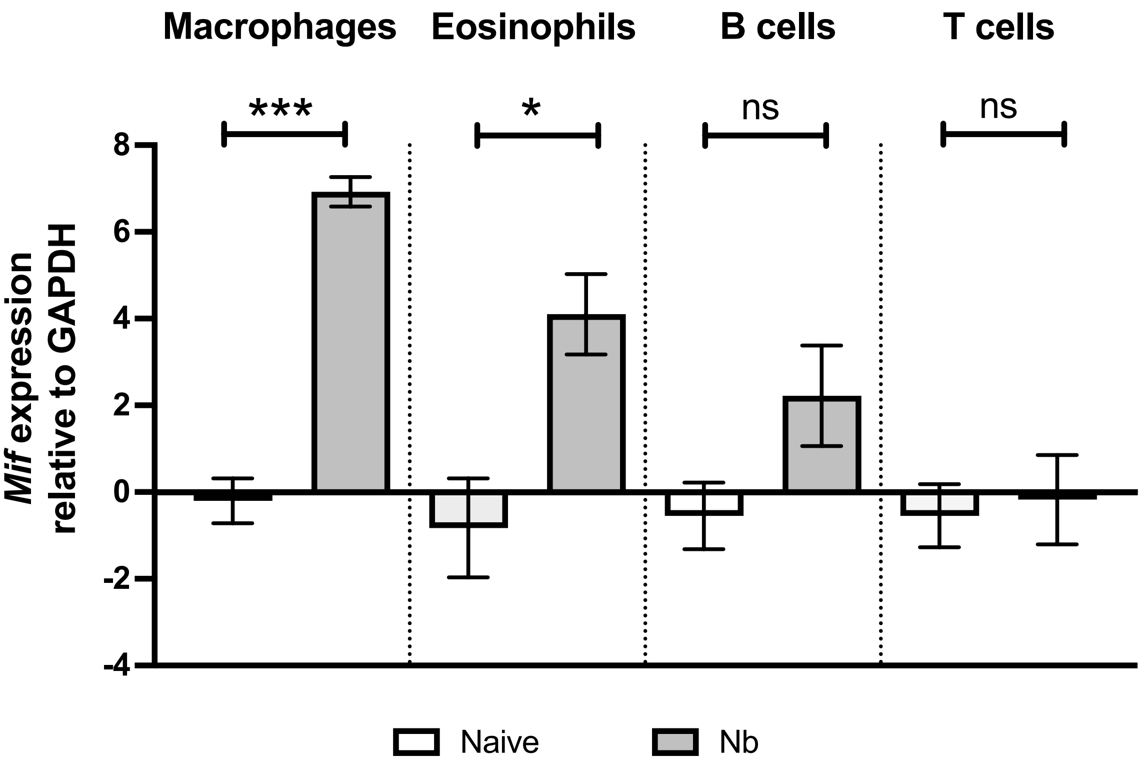

Supplementary Figure 5

***MIF* gene expression is significantly upregulated in macrophages and eosinophils following *N. brasiliensis* infection.**

BALB/c mice were infected with 400 L3 *N.brasiliensis* s.c. for 6 days and peritoneal exudate cells (PEC) were harvested and sorted by fluorescence-activated cell sorting (FACS) for gene expression analyses by qRT-PCR. *Mif* gene expression by sorted cells from naive and infected mice

A. F4/80^{+} CD11b^{+} Mφ

B. Siglec F^{+} eosinophils

C. CD19^{+}CD4^{-} B cells

D. CD19^{-}CD4^{+} T cells.

Graphs **A, B** are representative of two individually performed experiments with data from 3 individual mice), **C,D** show pooled data from two individually performed experiments (n=6 individual mice). Statistical analysis was performed using an unpaired Student's t test. * = p<0.05, *** p<0.001

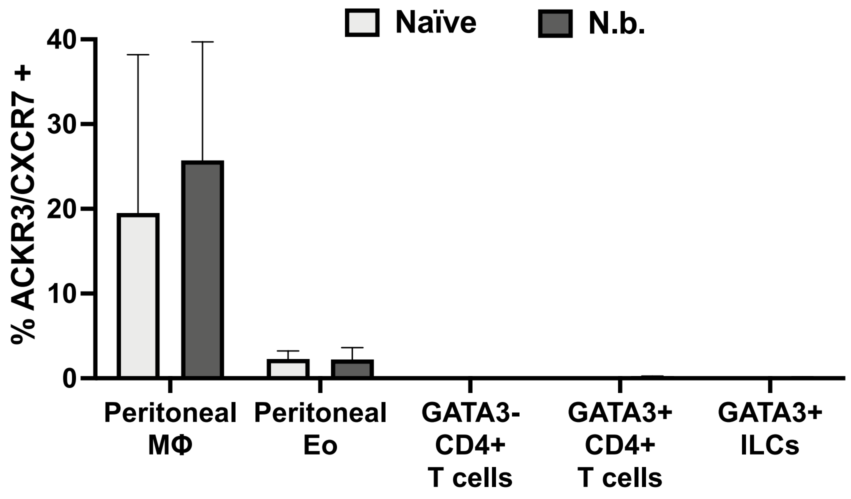

Supplementary Figure 6.
Peritoneal lavage and MLN cell ACKR3 (CXCR7) expression in *N.brasiliensis* infection.
Expression was measured by GFP levels in cell populations from naïve and day 6-infected GFP reporter mice; data are from a single experiment.

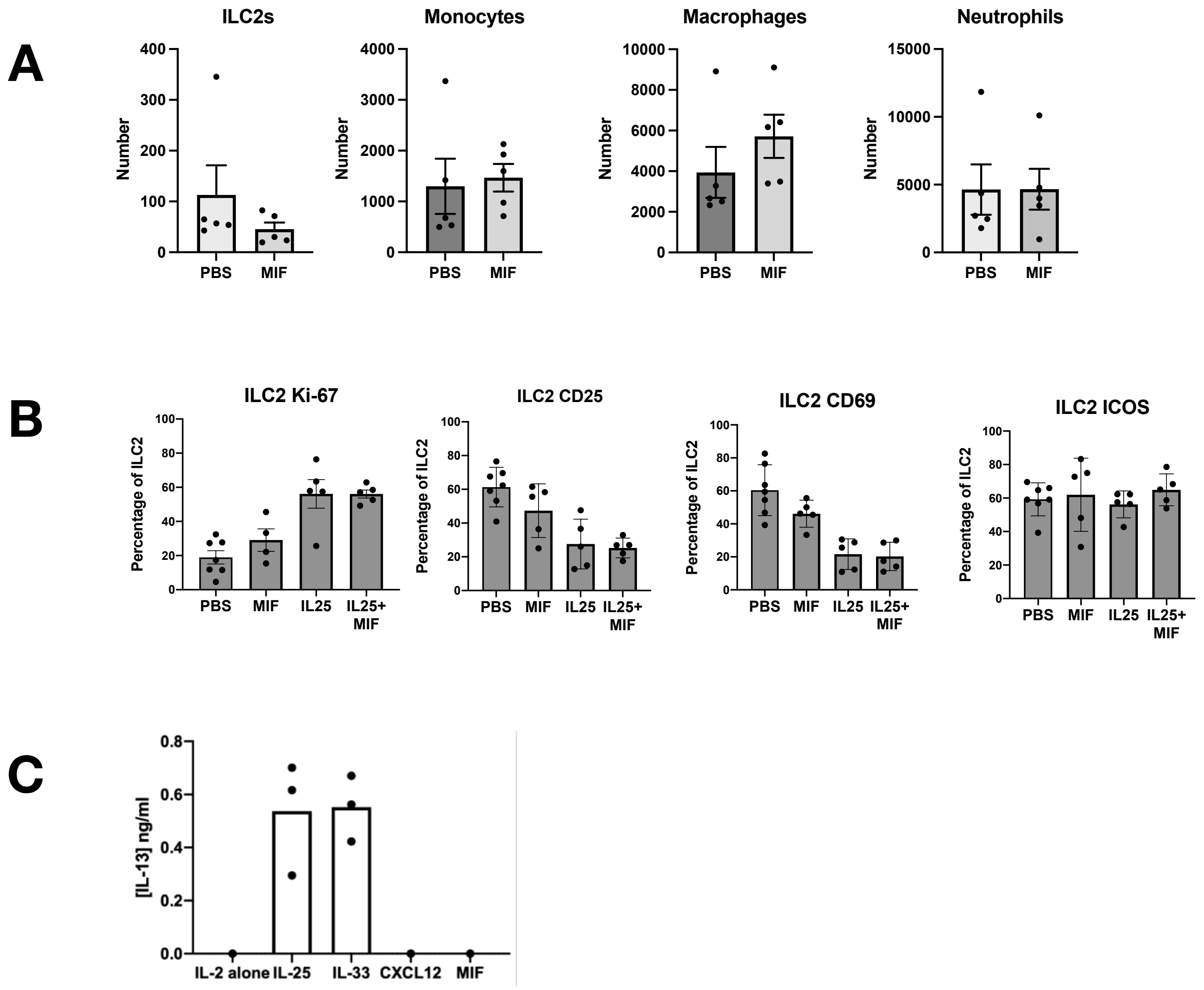

Supplementary Figure 7. Absence of direct activation of ILCs by MIF.

A. Peritoneal cell type numbers 48 hours following administration of MIF.

B. Peritoneal cell ILC2 populations harvested 48 hours following administration of MIF and/or IL-25, assayed by the indicated markers of cell proliferation (Ki67) and activation CD25, CD69, ICOS).

C. In vitro stimulation of *ex vivo* sorted ILC2s purified by MACS depletion from RAG-deficient mouse spleen, lymph node and peritoneal lavage, with MIF and/or IL-25

Suppl. Table 1. Primers for RT-PCR of Gene Expression

Primer	Protein	Primer sequence
DCLK1	Doublecortin-like kinase 1	F: CAG CCT GGA CGA GCT GGT GG<br>R: TGA CCA GTT GGG GTT CAC AT
GAPDH	Glyceraldehyde-3-phosphate dehydrogenase	F: ATG ACA TCA AGA AGG<br>R: CAT ACC AGG AAA TGA AAA TGA GTT G
GATA3	Transacting T-cell-specific transcription factor GATA-3	F: GGG TTC GGA TGT AAG TCG AG<br>R: CCA CAG TGG GGT AGA GGT TG
HPRT	Hypoxanthine phosphoribosyltransferase 1	F: AGG GAT TTG AAT CAC GTT TG<br>R: TTT ACT GGC AAC ATC AAC AG
IL-5	Interleukin 5	F: ACA TTG ACC GCC AAA AAG AG<br>R: ATC CAG GAA CTG CCT CGT
IL-25	Interleukin 25	F: TGG AGC TCT GCA TCT GTG TC<br>R: CGA TTC AAG TCC CTG TCC A
MIF	Macrophage migration inhibitory factor	F: ACA GCA TCG GCA AGA TCG<br>R: GGC CAC ACA GCA GCT TAC T
RETLNB	Resistin-like beta protein	F: CGT CTC CCT TTT CCC ACT G<br>R: CAG GAG ATC GTC TTA GGC TCT
